# Differences between children and adolescents in treatment response to atomoxetine and the correlation between health-related quality of life and Attention Deficit/Hyperactivity Disorder core symptoms: Meta-analysis of five atomoxetine trials

**DOI:** 10.1186/1753-2000-4-30

**Published:** 2010-12-06

**Authors:** Peter M Wehmeier, Alexander Schacht, Rodrigo Escobar, Nicola Savill, Val Harpin

**Affiliations:** 1 Lilly Deutschland GmbH, Medical Department, Bad Homburg, Germany; 2 Department of Child and Adolescent Psychiatry and Psychotherapy, Central Institute of Mental Health, Mannheim, University of Heidelberg, Germany; 3 European Medical Department, Eli Lilly & Co., Alcobendas, Spain; 4 Eli Lilly & Co., Basingstoke, UK; 5 Sheffield Children's NHS Foundation Trust, UK

## Abstract

**Objectives:**

To explore the influence of age on treatment responses to atomoxetine and to assess the relationship between core symptoms of attention deficit/hyperactivity disorder (ADHD) and health-related quality of life (HR-QoL) outcomes.

**Data Sources:**

Data from five similar clinical trials of atomoxetine in the treatment of children and adolescents with ADHD were included in this meta-analysis.

**Study Selection:**

Atomoxetine studies that used the ADHD Rating Scale (ADHD-RS) and the Child Health and Illness Profile Child Edition (CHIP-CE) as outcome measures were selected.

**Interventions:**

Treatment with atomoxetine.

**Main Outcome Measures:**

Treatment group differences (atomoxetine vs placebo) in terms of total score, domains, and subdomains of the CHIP-CE were compared across age groups, and correlations between ADHD-RS scores and CHIP-CE scores were calculated by age.

**Results:**

Data of 794 subjects (611 children, 183 adolescents) were pooled. At baseline, adolescents showed significantly (p < 0.05) greater impairment compared with children in the Family Involvement, Satisfaction with Self, and Academic Performance subdomains of the CHIP-CE. Treatment effect of atomoxetine was significant in both age groups for the Risk Avoidance domain and its subdomains. There was a significant age-treatment interaction with greater efficacy seen in adolescents in both the Risk Avoidance domain and the Threats to Achievement subdomain. Correlations between ADHD-RS and CHIP-CE scores were generally low at baseline and moderate in change from baseline and were overall similar in adolescents and children.

**Conclusions:**

Atomoxetine was effective in improving some aspects of HR-QoL in both age groups. Correlations between core symptoms of ADHD and HR-QoL were low to moderate.

## 1. Introduction

Attention deficit/hyperactivity disorder (ADHD) is one of the most frequently diagnosed psychiatric disorders in childhood, characterized by 3 core symptoms: inattentiveness, hyperactivity, and impulsivity. According to a recent meta-analysis[1], ADHD affects 5.29% of school-aged children worldwide. ADHD was consistently associated with complex short-term and long-term impairments and negative outcomes regarding educational achievement, social and emotional impairment, behavioral disturbances, problems with interpersonal relations, and psychiatric comorbidity [2-5].

The impact of ADHD goes beyond the direct effects of core symptoms on the individual's everyday functions and represents a serious burden on the patient's and the family's life, seriously impairing the emotional, social, and physical well-being of patients and hence their health-related quality of life (HR-QoL)[6].

HR-QoL has received increasing attention in children and adolescents with ADHD, both from clinicians and investigators [7-10]. HR-QoL is a multidimensional concept that reflects the subjective physical, social, and psychological aspects of health, and goes beyond symptoms of the disorder and objective functional outcomes [11].

Based on consistent findings in the literature, effective treatments exist for the management of ADHD with both pharmacotherapy and psychosocial interventions. The treatment options for ADHD include psychostimulants (e.g. methylphenidate, mixed amphetamine salts) or atomoxetine, which is a non-stimulant treatment option for ADHD [12], both in combination with behavioral therapy [13]. Atomoxetine is a selective norepinephrine reuptake inhibitor, and its efficacy and tolerability were demonstrated in a number of randomized, placebo-controlled trials among children and adolescents [14-17]. In addition, several studies have shown improvement of emotional well-being and HR-QoL in children and adolescents treated with atomoxetine [15,18-25]. As a non-controlled substance with no abuse liability, atomoxetine can be of value in certain populations such as patients with ADHD and co-morbid substance abuse disorder [26].

Although it has previously been thought that ADHD is essentially a disorder of childhood, a growing body of literature suggests that the disorder persists through adolescence and into adulthood with some core features and associated impairments still evident [2,7,27-29].

The clinical symptoms of ADHD change over time [3,28-32]. Specifically, hyperactive/impulsive symptoms generally decline, while inattentive symptoms might persist, or even become relatively more pronounced, taking into consideration the increased complexity of those cognitive tasks that a child or an adolescent is exposed to [3,30]. This is not surprising, as transition from childhood to adolescence involves a number changes that touch upon many areas of the adolescent's daily life. These changes include an increase in physical size and maturation, the desire to individuate from parents, resulting in more time spent away from home, an increase in the number of life activities to which the adolescent must adapt. Most of these changes are adversely affected by the delay in self-regulation that is usually associated with ADHD. Impaired self-esteem and sociability in adolescents is often the result. In adolescence, symptoms of inattention and impaired executive function (EF) generally have a greater impact on school functioning than the symptoms of hyperactivity and impulsivity. Impulsivity, in turn, is more related to functional impairment in non-academic domains and may be associated with the development of oppositional defiant disorder (ODD), drug experimentation, speeding while driving, engaging in risky sexual behavior, impulsive verbal behavior, and reactive aggression [5].

Thus, it is important to understand the implications for the individual as they get older and to evaluate medication effects with respect to age.

We therefore conducted a meta-analysis all atomoxetine clinical trials measuring HR-QoL using the Child Health and Illness Profile, Child Edition (CHIP-CE) Parent Edition that were in the Lilly data base to investigate the possible age effect on baseline impairments with regard to HR-QoL [8,33-35], and to explore the influence of age on treatment effects of atomoxetine regarding HR-QoL outcomes, in children (6-11 years) and adolescents (12-17 years) with ADHD. Additionally, we analyzed the correlation between ADHD core symptoms and HR-QoL at baseline, at endpoint, and for change from baseline in order to evaluate the association between the improvement of the core symptoms and the improvement of HR-QoL. Treatment effects were assessed based on the 3 placebo-controlled trials and correlations were examined leveraging all 5 studies found in the Lilly data base.

## 2. **Methods**

### 2.1 Studies included in the meta-analysis

Data from 5 atomoxetine clinical trials (4 from Europe, 1 from Canada) with similar inclusion and exclusion criteria and similar duration of treatment (8-12 weeks follow-up) were included in the meta-analysis [23,36-39]. The total number of patients was 794. Design, sample size, and duration of the respective studies are described in Table 1.

**Table 1:** Basic information on the 5 clinical trials included in this meta-analysis

Study	Sample size (n)	Design	Duration	Dose mg/kg/day	Procedure
Study 1 (S) Svanborg et al, 2009 [36]	99	Randomized, double-blind, placebo-controlled	10 weeks	1.2	Diagnosis based on ADHD-RS, confirmed with KSADS, stimulant-naïve patients No ongoing psychotropic medication or structured PT
Study 2 (E) Escobar et al, 2009 [37]	149	Randomized, double-blind, placebo-controlled	12 weeks	1.2	Diagnosis based on ADHD-RS, confirmed with KSADS stimulant-naïve patients No ongoing psychotropic medication or structured PT CGI≥4 at inclusion
Study 3 (I) Dell'Agnello et al, 2007 [38]	139	Randomized, double-blind, placebo-controlled	8 weeks	1.2	Diagnosis based on ADHD-RS, confirmed with KSADS, ADHD+ODD patients No ongoing psychotropic medication or structured PT CGI≥4 at inclusion
Study 4 (UK) Prasad et al, 2007 [23]	201	Open-label, atomoxetine vs standard of care	10 weeks	0.5-1.8	Diagnosis based on ADHD-RS, confirmed with KSADS No ongoing psychotropic medication or structured PT
Study 5 (CAN) Dickson et al, 2007 [39]	206	Open-label, atomoxetine only	12 weeks	0.5-1.4	Diagnosis based on ADHD-RS, confirmed with KSADS

Abbreviations: ADHD-RS, Attention Deficit/Hyperactivity Disorder Rating Scale; KSADS, Kiddie Schedule for Affective Disorders and Schizophrenia for School Age Children; PT, psychotherapy; CGI, Clinical Global Impression; ODD, oppositional defiant disorder; S, Sweden; E, Spain; I, Italy; UK, United Kingdom; CAN, Canada

All included patients met the Diagnostic and Statistical Manual of Mental Disorders, Fourth Edition (DSM-IV) [40] diagnostic criteria for ADHD and had a symptom severity of at least 1.5 standard deviations (SD) above the normative values of the Attention Deficit/Hyperactivity Disorder Rating Scale-IV, (ADHD-RS) Parent Version [41] except for Study 3, where the ADHD subscale of the SNAP (Swanson, Nolan, and Pelham-IV) [42] was applied. In all studies, except in Study 5, the diagnosis was confirmed using the Kiddie Schedule for Affective Disorders and Schizophrenia for School Age Children-Present and Lifetime Version (K-SADS-PL) [43], a semistructured diagnostic interview that includes a supplement for ADHD. In studies 2 and 3, baseline Clinical Global Impression of Severity (CGI-S) [44] scores for ADHD were at least 4 or higher.

Studies 1 and 2 recruited only stimulant-naïve patients. Study 3, which was carried out in Italy, did not explicitly require medication-naïve patients, but at the time of recruitment, there were no ADHD drugs approved by authorities in that country.

### 2.2 Measures

#### 2.2.1 CHIP-CE

The primary scale on which this meta-analysis was based is the CHIP-CE Parent Report Form [33,34], a 76-item generic HR-QoL questionnaire, covering a total of 5 domains (Satisfaction, Comfort, Risk Avoidance, Resilience, and Achievement) and 12 subdomains (Satisfaction with Health [SH], Satisfaction with Self [SS], Physical Comfort [PC], Emotional Comfort [EC], Restricted Activity [RA], Individual Risk Avoidance [IRA], Threats to Achievement [TA], Family Involvement [FI], Physical Activity [PA], Social Problem Solving [SPS], Academic Performance [AP], and Peer Relations [PR]). Table 2 explains which aspects of HR-QoL are assessed by each domain of the CHIP-CE. More recently, a CHIP-CE total score has been developed, which can be used as a global measure of HR-QoL [35].

**Table 2:** CHIP-CE: Parent Report Form (PRF) Domain and Subdomain Definitions

CHIP-CE domains and subdomains	Definition
Satisfaction Domain	The parent's assessment of the child's sense of well-being and self-esteem (11 items)
*Satisfaction with health*	Overall perceptions of well-being and health
*Self-esteem*	General self-concept
Comfort Domain	Parent's assessment of the child's experience of physical and emotional symptoms and positive health sensations and observed limitations of activity (22 items)
*Physical comfort*	Positive and negative somatic feelings and symptoms
*Emotional comfort*	Positive and negative emotional feelings and symptoms
*Restricted activity*	Restrictions in day-to-day activities due to illness
Resilience Domain	Parent's perception of the child's participation in family, coping abilities and physical activity (19 items)
*Family involvement*	Level of activities with family and perceived family support
*Social problem-solving*	Active approaches to solving an interpersonal problem
*Physical activity*	Level of involvement in activities related to fitness
Risk Avoidance Domain	Degree to which parent perceives that the child avoids behaviors that increase the likelihood of illness, injury, or poor social development (14 items)
*Individual risk avoidance*	Avoidance of activities that threaten individual health and development
*Threats to achievement*	Avoidance of behaviors that typically disrupt social development
Achievement Domain	Extent to which the parent perceives that the child meets expectations for role performance in school and with peers (10 items)
*Academic performance*	School performance and engagement
*Peer relations*	Relationships with peer group

The structure of the CHIP-CE was developed in non-ADHD samples. The CHIP-CE scores are standardized to t scores with a mean (± SD) of 50 (± 10), with higher scores indicating better health. Normative data were derived from a sample of 1049 school-aged children from the United States [33,34].

#### 2.2.2 ADHD-RS

The evaluation of the treatment effect of atomoxetine on core ADHD symptoms was based on the ADHD-RS [41], which evaluates all 18 symptoms of ADHD according to the DSM-IV diagnostic criteria. Improvement is indicated by a decrease in the score. The ADHD-RS comprises a total score, an inattentive sub-score, and a hyperactive/impulsive sub-score.

### 2.3 Statistical analysis

The demographic and baseline data were summarized by descriptive statistics unadjusted for study. Group comparisons at baseline were based on two-way analysis of variance (ANOVA) using the terms age and study for continuous variables and based on the Cochran-Mantel-Haenszel test controlling for study in the case of categorical variables.

Treatment efficacy over time was analyzed on an intent-to-treat basis. The intent-to-treat population included patients who had been randomized, had a baseline observation, and at least one postbaseline observation. The last observation was the one reported for change from baseline. Treatment-group differences were compared using a fixed effect analysis of covariance (ANCOVA) model including the terms treatment, study, age group, baseline ADHD-RS score, and the respective baseline CHIP-CE score. The model was run for a second time with the treatment-by-age subgroup interaction term added. Effect size (Cohen's d) was calculated for treatment overall and within age subgroups. Effect size was calculated as the ratio of the difference between atomoxetine and placebo at endpoint divided by the standard deviation of the residuals.

A consistent treatment effect in the groups is stated, if the overall treatment effect is significant and the effect sizes are clinically similar in both age groups.

Correlations between ADHD-RS scores (total score, inattentive, and hyperactive/impulsive sub-scores) and CHIP-CE scores (total, domain, and sub-domain scores) at baseline, at endpoint, and for the change from baseline to endpoint, are shown by age subgroup using Pearson's correlation coefficient and the corresponding 95% confidence interval.

All tests of hypotheses were considered statistically significant if the two-sided p-value was < 0.05. An alpha level of 0.10 was used to judge the statistical significance of an interaction. No correction was done for multiple testing as this is a post hoc analysis on existing data. The Statistical Analysis System (version 9; SAS Institute, Cary NC) was used for all analyses.

## 3. Results

### 3.1 Patient disposition

Data from a total of 794 patients were included in the analysis. The age range was 6 to 15 years. The mean age was 9.7 years (SD 2.30 years). Most of the patients of the pooled sample were children (< 12 years): 611 (77.0%), and male 658 (82.9%). For the evaluation of the effect of atomoxetine on HR-QoL, as measured by the CHIP-CE, samples from only the placebo-controlled trials were included. In total, data of n = 183 and n = 92 children (6-11 years) and n = 72 and n = 40 adolescents (12-17 years) were analyzed in the atomoxetine and placebo groups, respectively. For the comparison of the correlations between core ADHD symptoms and HR-QoL, across age groups, we included the data of all studies in the analyses. Demographic data of the pooled sample are summarized in Table 3.

**Table 3:** Demographic and baseline data of the pooled sample

	Placebo-controlled studies			All studies		
**Characteristics**	**Children** **(N = 275)**	**Adolescents** **(N = 112)**	**p-value**	**Children** **(N = 611)**	**Adolescents** **(N = 183)**	**p-value**

**Gender**			0.45			0.152
Female (n, %)	44 (16.0)	16 (14.3)		115 (18.8)	21 (11.5)	
Male (n, %)	231 (84.0)	96 (85.7)		496 (81.2)	162 (88.5)	
**Age, mean (SD), y**	8.7 (1.53)	13.0 (1.04)	NA	8.7 (1.51)	13.0 (0.99)	NA
**ADHD subtype**			0.002			<0.001
Combined (n, %)	223 (81.1)	72 (64.3)		508 (83.1)	133 (72.7)	
Hyperactive/impulsive (n, %)	10 (3.6)	7 (6.3)		14 (2.3)	10 (5.5)	
Inattentive (n, %)	42 (15.3)	33 (29.5)		89 (14.6)	40 (21.9)	
**ADHD-RS, mean (SD)**						
Total score	41.4 (7.42)	38.4 (7.83)	0.002	42.1 (7.87)	41.0 (8.57)	0.004
Inattentive subscore	21.6 (3.70)	21.9 (3.85)	0.35	22.1 (3.80)	22.5 (3.90)	0.374
Hyperactive/impulsive subscore	19.8 (5.51)	16.5 (6.53)	<0.001	20.0 (5.79)	18.4 (6.67)	<0.001

p-value based on two-way analysis of variance (ANOVA) including terms age and study for continuous variables and based on Cochran-Mantel-Haenszel test controlling for study for categorical variables

Abbreviations: SD, standard deviation; ADHD, attention deficit/hyperactivity disorder; ADHD-RS, Attention Deficit/Hyperactivity Disorder Rating Scale; NA, Not Applicable

### 3.2 Baseline differences across age groups

In the population of the five studies, gender distribution was similar across age groups. The proportion of ADHD combined subtype according to DSM-IV was significantly higher and, accordingly, the proportion of the inattentive subtype was significantly lower in children compared with adolescents. This difference was also reflected in the ADHD-RS scores, where the hyperactive/impulsive subscore was significantly higher in children, leading to a significantly higher total score (Table 3).

Impaired HR-QoL was observed at baseline as the CHIP-CE total score and four of the five domain scores (Table 4) had means of less than 40. Impairments in the following sub-domains were observed (mean <40 for at least one group - all studies): Satisfaction with Self, Emotional Comfort, Individual Risk Avoidance, Threats to Achievement, Family Involvement, Social Problem Solving, Academic Performance, and Peer Relations. Adolescents were significantly more impaired at baseline in the Satisfaction with Self and the Family Involvement sub-domains as well as in the Achievement domain and the Academic Performance sub-domain. On the other hand, children were significantly more impaired at baseline in the Emotional Comfort sub-domain. The Restricted Activity sub-domain showed a significant difference between children and adolescents; however, mean and SD in this sub-domain were within the normal range, indicating relevant impairment neither in children nor in adolescents. Although the Individual Risk Avoidance sub-domain score showed a statistically significant difference between adolescents and children in the analysis adjusting for study (p = 0.004), unadjusted descriptive scores did not indicate a clinically relevant difference (mean = 35.6, SD = 15.71 for children; mean = 35.8, SD = 15.28 for adolescents).

**Table 4:** Child Health and Illness Profile-Child Edition, baseline data

	Placebo controlled studies			All studies		
**CHIP-CE items**	**Children** **(N = 275)**	**Adolescents** **(N = 112)**	**p-value**	**Children** **(N = 611)**	**Adolescents** **(N = 183)**	**p-value**

**CHIP-CE**						
**Total Score**	31.9 (10.87)	29.3 (11.80)	0.030	29.3 (11.58)	27.5 (12.29)	0.296
**Satisfaction Domain**	36.3 (13.66)	32.9 (14.20)	0.031	34.9 (13.88)	32.9 (14.49)	0.066
Satisfaction with Health	43.0 (12.89)	40.8 (14.13)	0.294	40.9 (13.22)	40.6 (14.45)	0.388
Satisfaction with Self	32.4 (13.99)	28.6 (13.88)	**0.004**	32.3 (14.34)	29.0 (14.21)	**0.018**
**Comfort Domain**	46.0 (9.92)	46.2 (10.35)	0.526	43.3 (10.75)	44.7 (11.00)	0.426
Physical Comfort	52.0 (9.32)	52.7 (9.80)	0.423	50.7 (9.84)	52.0 (10.18)	0.850
Emotional Comfort	41.0 (10.96)	42.0 (10.76)	0.162	37.7 (11.80)	39.9 (11.59)	**0.029**
Restricted Activity	50.5 (10.13)	47.4 (11.07)	**0.022**	50.2 (10.02)	48.1 (10.89)	**0.027**
**Risk Avoidance Domain**	33.7 (12.11)	32.8 (12.54)	0.396	30.6 (14.75)	29.0 (14.18)	0.378
Individual Risk Avoidance	39.1 (13.15)	40.9 (12.83)	0.213	35.6 (15.71)	35.8 (15.28)	**0.004**
Threats to Achievement	33.8 (11.80)	31.8 (12.39)	0.064	31.4 (13.67)	29.2 (13.27)	0.719
**Resilience Domain**	36.2 (11.98)	35.2 (11.20)	0.197	36.5 (11.91)	34.5 (12.33)	0.096
Family Involvement	40.6 (10.84)	36.7 (10.74)	**<0.001**	41.4 (11.26)	36.3 (12.24)	**<0.001**
Physical Activity	44.4 (11.20)	45.2 (11.04)	0.952	46.4 (11.73)	46.5 (11.94)	0.373
Social Problem Solving	36.9 (12.58)	38.2 (12.36)	0.452	35.1 (13.01)	35.8 (12.85)	0.112
**Achievement Domain**	33.4 (9.92)	29.2 (10.50)	**<0.001**	31.0 (10.26)	28.9 (10.71)	**0.046**
Academic Performance	32.8 (9.47)	27.9 (8.80)	**<0.001**	32.0 (9.91)	27.7 (9.38)	**<0.001**
Peer Relations	39.7 (13.35)	38.6 (14.40)	0.703	36.7 (13.19)	38.4 (14.12)	0.051

Data is presented as unadjusted mean and SD (if not otherwise indicated)

p-value is based on two-way analysis of variance (ANOVA) including terms age and study

Abbreviations: CHIP-CE, Child Health and Illness Profile, Child Edition; SD, standard deviation

Significant p-values are bolded

### 3.3 Treatment effect of atomoxetine

**Figure 1:**
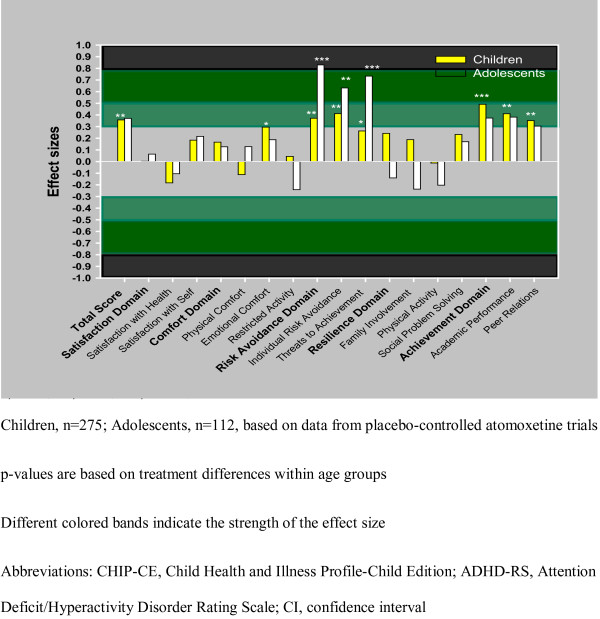
**Figure 1 shows the effect sizes of atomoxetine in improving CHIP-CE scores, by age groups, based on data of the 3 placebo-controlled trials**. P values are based on treatment differences within age groups and are shown by astericks, as follows: *p≤0.05; **p≤0.01; ***p≤0.001.

The treatment effect of atomoxetine as reflected by the CHIP-CE was significant overall and consistent within both age groups for the total score, the Emotional Comfort sub-domain, and for the Achievement domain with its two sub-domains Academic Performance and Peer Relations. In the Risk Avoidance domain, there was a significant age interaction with the therapeutic effect of atomoxetine (p < 0.10). This interaction was due to the significant interaction found in the Threats to Achievement sub-domain (p < 0.10). Specifically, in the Risk Avoidance domain and in its two sub-domains (IRA, TA), effect sizes indicated a more pronounced therapeutic effect of atomoxetine for adolescents compared with children (see Table 5, 6 and Figure 1).

**Table 5:** Child Health and Illness Profile-Child Edition, change from baseline based on data of the 3 placebo-controlled trials.

	Children (n = 275)		Adolescents (n = 112)	
**CHIP-CE items** Mean change (SE)	**Atomoxetine (n = 183)**	**Placebo (n = 92)**	**Atomoxetine (n = 72)**	**Placebo (n = 40)**

**Total Score**	4.60 (0.62)	2.06 (0.80)	5.40 (0.94)	2.48 (1.28)
**Satisfaction Domain**	2.11 (0.83)	2.18 (1.07)	2.88 (1.22)	2.19 (1.65)
Satisfaction With Health	0.40 (0.75)	2.06 (0.97)	1.29 (1.21)	2.57 (1.65)
Satisfaction With Self	3.45 (0.88)	1.84 (1.14)	3.84 (1.28)	1.21 (1.73)
**Comfort Domain**	2.47 (0.65)	1.34 (0.84)	2.57 (0.93)	1.33 (1.26)
Physical Comfort	0.68 (0.64)	1.53 (0.83)	0.91 (0.81)	-0.01 (1.10)
Emotional Comfort	3.37 (0.76)	1.01 (0.99)	3.34 (0.97)	1.23 (1.31)
Restricted Activity	0.81 (0.67)	0.49 (0.88)	0.46 (1.20)	2.20 (1.63)
**Risk Avoidance Domain**	4.63 (0.64)	1.90 (0.82)	7.27 (0.93)	0.70 (1.28)
Individual Risk Avoidance	4.16 (0.67)	0.82 (0.86)	4.59 (1.15)	-0.95 (1.56)
Threats to Achievement	4.08 (0.66)	2.12 (0.84)	7.35 (0.98)	1.53 (1.35)
**Resilience Domain**	3.20 (0.68)	1.23 (0.88)	1.00 (0.97)	1.69 (1.32)
Family Involvement	1.69 (0.70)	0.18 (0.91)	0.19 (1.00)	2.05 (1.35)
Physical Activity	1.08 (0.72)	1.16 (0.92)	-2.15 (1.10)	-0.42 (1.49)
Social Problem Solving	3.54 (0.77)	1.31 (0.99)	3.26 (1.12)	1.67 (1.56)
**Achievement Domain**	4.04 (0.60)	0.68 (0.79)	4.83 (0.86)	2.13 (1.21)
Academic Performance	4.03 (0.68)	1.01 (0.88)	5.21 (0.91)	2.00 (1.28)
Peer Relations	2.61 (0.57)	0.30 (0.74)	2.76 (0.80)	0.52 (1.09)

All values are presented as LS Means (SE)

Abbreviations: CHIP-CE, Child Health and Illness Profile-Child Edition; SE, standard error; LS, least square

**Table 6:** Effect sizes (Cohen's d) of atomoxetine for improving Child Health and Illness Profile-Child Edition scores based on data of the 3 placebo-controlled trials.

	Children		Adolescents		Interaction^a^	Overall	
CHIP-CE domains and sub-domains	Effect size	p-value	Effect size	p-value	p-value	Effect size	p-value
**Total Score**	0.357	**0.007**	0.370	0.068	0.957	0.353	**0.002**
**Satisfaction Domain**	0.002	0.988	0.063	0.757	0.801	0.024	0.829
Satisfaction with Health	-0.183	0.169	-0.105	0.606	0.746	-0.159	0.157
Satisfaction with Self	0.183	0.168	0.216	0.287	0.892	0.198	0.080
**Comfort Domain**	0.166	0.213	0.126	0.534	0.869	0.162	0.150
Physical Comfort	-0.112	0.402	0.127	0.532	0.322	-0.026	0.820
Emotional Comfort	0.295	**0.027**	0.187	0.357	0.653	0.268	**0.018**
Restricted Activity	0.044	0.742	-0.242	0.245	0.246	-0.032	0.777
**Risk Avoidance Domain**	0.371	**0.005**	0.829	**<0.001**	**0.059**	0.489	**<0.001**
Individual Risk Avoidance	0.411	**0.002**	0.631	**0.002**	0.361	0.463	**<0.001**
Threats to Achievement	0.262	**0.050**	0.733	**<0.001**	**0.053**	0.387	**<0.001**
**Resilience Domain**	0.242	0.069	-0.141	0.487	0.112	0.131	0.247
Family Involvement	0.188	0.158	-0.237	0.243	0.078	0.055	0.627
Physical Activity	-0.013	0.921	-0.203	0.315	0.430	-0.062	0.581
Social Problem Solving	0.232	0.083	0.170	0.409	0.799	0.219	0.054
**Achievement Domain**	0.491	**<0.001**	0.373	0.078	0.637	0.431	**<0.001**
Academic Performance	0.410	**0.003**	0.381	0.072	0.909	0.376	**0.001**
Peer Relations	0.351	**0.008**	0.304	0.134	0.845	0.316	**0.005**

Abbreviations: CHIP-CE, Child Health and Illness Profile-Child Edition

Significant p-values are bolded; ^a^interaction p-values indicate the possible age-effect

### 3.4 Correlations between ADHD-RS and CHIP-CE scores

**Figure 2:**
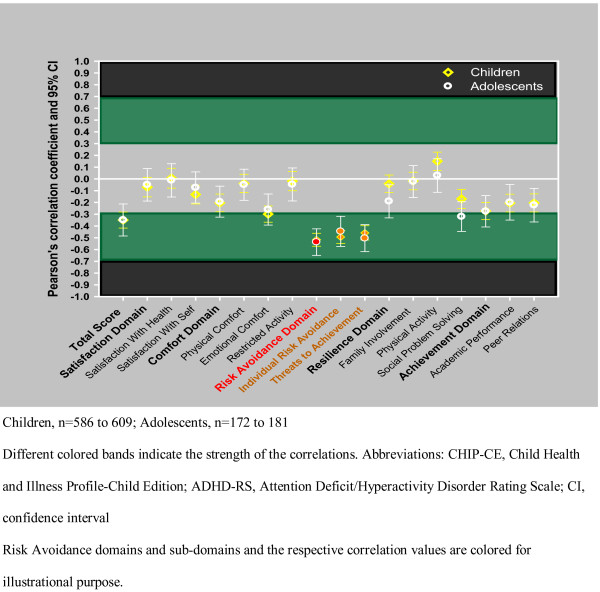
**Figure 2 shows the Pearson's correlation coefficients between the CHIP-CE baseline score and ADHD-RS total score, by age groups based on data of all 5 trials**. Colored text and dots are used for illustrational purposes.

**Figure 3:**
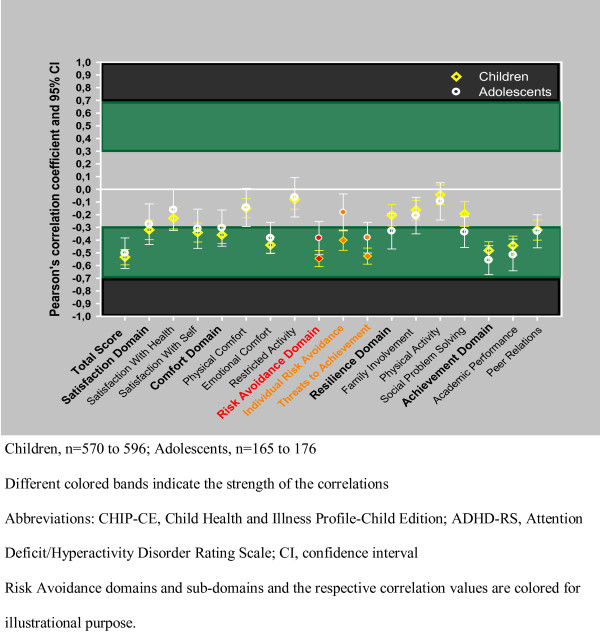
**Figure 3 shows the Pearson's correlation coefficients between the CHIP-CE change from baseline and ADHD-RS total score, by age groups based on data of all 5 trials**. Colored text and dots are used for illustrational purposes in case of those subdomains where remarkable change can be detected compared with baseline values.

The correlation values with the 95% CI are summarized in Table 7, 8, 9 and Figure 2 and 3, by age groups. The CHIP-CE scores and the ADHD-RS scores showed consistent negative correlations at baseline, endpoint, and in change from baseline. Negative correlations indicate that patients with high ADHD-RS scores have low CHIP-CE scores and vice versa. Overall, correlations were in the small to medium range, showing a consistent trend toward stronger correlations at endpoint and in change from baseline, compared with the baseline correlations. In general, correlations were consistently the strongest for the Risk Avoidance and Achievement domains and their sub-domains, while correlations were consistently the weakest for the Satisfaction domain and sub-domains. The relatively strong correlation between the Risk Avoidance domain and ADHD-RS total score was predominantly influenced by the correlation with the hyperactive/impulsive ADHD-RS sub-score, while the inattentive sub-score exerted greater influence on the correlations between the Achievement domain and the ADHD-RS scores.

**Table 7:** Correlation between Child Health and Illness Profile-Child Edition and ADHD-Rating Scale total score, by age groups based on data of all 5 trials.

	Baseline			Endpoint			Change from baseline		
CHIP-CE	n	r	95% CI	N	r	95% CI	n	r	95% CI
*Children*									
**Total Score**	609	-0.350	-0.418 to -0.282	598	-0.527	-0.589 to -0.466	596	-0.534	-0.595 to -0.474
**Satisfaction Domain**	604	-0.070	-0.153 to 0.013	598	-0.250	-0.327 to -0.174	591	-0.319	-0.396 to -0.242
Satisfaction with Health	604	0.006	-0.079 to 0.090	598	-0.153	-0.231 to -0.075	591	-0.228	-0.313 to -0.144
Satisfaction with Self	604	-0.133	-0.213 to -0.053	598	-0.305	-0.380 to -0.229	591	-0.340	-0.415 to -0.266
**Comfort Domain**	609	-0.204	-0.279 to -0.129	598	-0.301	-0.374 to -0.228	596	-0.359	-0.426 to -0.292
Physical Comfort	609	-0.039	-0.117 to 0.039	598	-0.099	-0.175 to -0.022	596	-0.149	-0.224 to -0.074
Emotional Comfort	609	-0.299	-0.368 to -0.230	598	-0.397	-0.468 to -0.032	596	-0.439	-0.503 to -0.375
Restricted Activity	586	-0.019	-0.101 to 0.062	594	-0.068	-0.148 to 0.011	570	-0.080	-0.157 to -0.003
**Risk Avoidance Domain**	608	-0.517	-0.572 to -0.462	598	-0.591	-0.649 to -0.533	595	-0.545	-0.608 to -0.482
Individual Risk Avoidance	609	-0.494	-0.548 to -0.439	597	-0.478	-0.545 to -0.411	595	-0.401	-0.481 to -0.321
Threats to Achievement	607	-0.459	-0.519 to -0.398	598	-0.571	-0.628 to -0.514	594	-0.526	-0.590 to -0.463
**Resilience Domain**	609	-0.042	-0.116 to 0.033	597	-0.284	-0.361 to -0.208	595	-0.205	-0.289 to -0.120
Family Involvement	609	-0.018	-0.093 to 0.057	597	-0.195	-0.272 to -0.118	595	-0.163	-0.240 to -0.087
Physical Activity	609	0.150	0.072 to 0.227	597	-0.103	-0.183 to -0.023	595	-0.043	-0.120 to 0.034
Social Problem Solving	606	-0.170	-0.251 to -0.089	597	-0.261	-0.341 -0.180	592	-0.193	-0.292 to -0.095
**Achievement Domain**	598	-0.273	-0.345 to -0.201	590	-0.467	-0.535 to -0.399	579	-0.482	-0.550 to -0.413
Academic Performance	598	-0.206	-0.281 to -0.130	589	-0.449	-0.521 to -0.378	578	-0.443	-0.517 to -0.369
Peer Relations	607	-0.204	-0.281 to -0.127	598	-0.288	-0.363 to -0.213	594	-0.321	-0.400 to -0.242
***Adolescents***									
**Total Score**	181	-0.349	-0.485 to -0.213	177	-0.535	-0.637 to -0.434	176	-0.503	-0.624 to -0.383
**Satisfaction Domain**	181	-0.050	-0.188 to 0.088	177	-0.203	-0.345 to -0.061	176	-0.275	-0.435 to -0.115
Satisfaction with Health	180	-0.012	-0.154 to 0.129	177	-0.127	-0.267 to 0.013	176	-0.162	-0.324 to -0.000
Satisfaction with Self	181	-0.074	-0.206 to 0.059	177	-0.239	-0.382 to -0.096	176	-0.310	-0.465 to -0.156
**Comfort Domain**	180	-0.194	-0.325 to -0.062	177	-0.289	-0.430 to -0.148	175	-0.305	-0.448 to -0.163
Physical Comfort	180	-0.050	-0.182 to 0.082	177	-0.064	-0.208 to 0.080	176	-0.143	-0.293 to 0.007
Emotional Comfort	179	-0.260	-0.392 to -0.128	177	-0.399	-0.526 to -0.272	174	-0.384	-0.505 to -0.262
Restricted Activity	172	-0.047	-0.187 to 0.093	173	-0.107	-0.243 to 0.029	165	-0.063	-0.218 to 0.093
**Risk Avoidance Domain**	180	-0.537	-0.650 to -0.424	176	-0.567	-0.685 to -0.449	174	-0.384	-0.515 to -0.254
Individual Risk Avoidance	180	-0.446	-0.574 to -0.318	177	-0.424	-0.571 to -0.277	175	-0.182	-0.327 to -0.037
Threats to Achievement	180	-0.504	-0.618 to -0.389	176	-0.570	-0.675 to -0.466	174	-0.381	-0.502 to -0.261
**Resilience Domain**	180	-0.190	-0.331 to -0.050	177	-0.290	-0.419 to -0.162	175	-0.329	-0.470 to -0.188
Family Involvement	179	-0.023	-0.158 to 0.113	177	-0.171	-0.306 to -0.036	174	-0.207	-0.351 to -0.063
Physical Activity	179	0.029	-0.114 to 0.171	177	0.003	-0.137 to 0.142	175	-0.095	-0.242 to 0.051
Social Problem Solving	180	-0.320	-0.446 to -0.194	176	-0.388	-0.516 to -0.259	174	-0.337	-0.458 to -0.216
**Achievement Domain**	176	-0.275	-0.408 to -0.142	171	-0.562	-0.661 to -0.462	166	-0.558	-0.672 to -0.444
Academic Performance	175	-0.199	-0.350 to -0.048	171	-0.610	-0.705 to -0.515	165	-0.517	-0.642 to -0.392
Peer Relations	180	-0.223	-0.366 to -0.080	177	-0.296	-0.436 to -0.156	175	-0.331	-0.461 to -0.201

Abbreviations: CHIP-CE, Child Health and Illness Profile-Child Edition; ADHD-RS, Attention Deficit/Hyperactivity Disorder Rating Scale; CI, confidence interval; r, Pearson's correlation coefficient

**Table 8:** Correlation between Child Health and Illness Profile-Child Edition and ADHD-Rating Scale inattentive subscore, by age groups based on data of all 5 trials.

CHIP-CE	Baseline		Endpoint		Change from baseline	
	R	95% CI	r	95% CI	r	95% CI
*Children n = 570-609*						
**Total Score**	-0.275	-0.345 to -0.205	**-0.513**	-0.575 to -0.452	**-0.535**	-0.595 to -0.475
**Satisfaction Domain**	-0.137	-0.213 to -0.060	-0.285	-0.361 to -0.209	**-0.327**	-0.403 to -0.252
Satisfaction with Health	-0.106	-0.183 to -0.028	-0.201	-0.278 to -0.124	-0.243	-0.326 to -0.159
Satisfaction with Self	-0.134	-0.210 to -0.058	**-0.315**	-0.390 to -0.240	**-0.340**	-0.412 to -0.268
**Comfort Domain**	-0.188	-0.260 to -0.116	**-0.317**	-0.391 to -0.243	**-0.354**	-0.423 to -0.285
Physical Comfort	-0.062	-0.141 to 0.016	-0.152	-0.230 to -0.073	-0.153	-0.227 to -0.079
Emotional Comfort	-0.240	-0.310 to -0.169	**-0.376**	-0.448 to -0.305	**-0.430**	-0.495 to -0.365
Restricted Activity	-0.068	-0.145 to 0.010	-0.095	-0.174 to -0.017	-0.078	-0.161 to 0.004
**Risk Avoidance Domain**	-0.273	-0.343 to -0.204	**-0.496**	-0.562 to -0.431	**-0.511**	-0.576 to -0.446
Individual Risk Avoidance	-0.293	-0.360 to -0.226	**-0.390**	-0.462 to -0.317	**-0.372**	-0.452 to -0.292
Threats to Achievement	-0.222	-0.293 to -0.151	**-0.486**	-0.550 to -0.422	**-0.497**	-0.562 to -0.431
**Resilience Domain**	-0.037	-0.117 to 0.042	-0.278	-0.355 to -0.201	-0.224	-0.307 to -0.142
Family Involvement	0.003	-0.077 to 0.084	-0.190	-0.266 to -0.113	-0.181	-0.256 to -0.106
Physical Activity	0.045	-0.036 to 0.125	-0.140	-0.220 to -0.060	-0.063	-0.140 to 0.014
Social Problem Solving	-0.104	-0.187 to -0.022	-0.227	-0.309 to -0.145	-0.199	-0.293 to -0.105
**Achievement Domain**	-0.267	-0.336 to -0.199	**-0.472**	-0.539 to -0.405	**-0.499**	-0.565 to -0.433
Academic Performance	-0.292	-0.362 to -0.221	**-0.493**	-0.561 to -0.425	**-0.463**	-0.536 to -0.390
Peer Relations	-0.101	-0.180 to -0.022	-0.245	-0.321 to -0.169	**-0.322**	-0.399 to -0.244
***Adolescents n = 165-181***						
**Total Score**	-0.175	-0.304 to -0.045	**-0.510**	-0.615 to -0.405	**-0.499**	-0.626 to -0.372
**Satisfaction Domain**	-0.040	-0.167 to 0.086	-0.237	-0.376 to -0.097	-0.291	-0.449 to -0.132
Satisfaction with Health	-0.040	-0.171 to 0.091	-0.157	-0.298 to -0.017	-0.191	-0.356 to -0.027
Satisfaction with Self	-0.028	-0.154 to 0.099	-0.268	-0.405 to -0.130	-0.308	-0.463 to -0.153
**Comfort Domain**	-0.070	-0.201 to 0.061	-0.235	-0.382 to -0.087	-0.282	-0.436 to -0.129
Physical Comfort	-0.027	-0.163 to 0.109	-0.054	-0.195 to 0.088	-0.137	-0.290 to 0.016
Emotional Comfort	-0.055	-0.192 to 0.082	**-0.309**	-0.451 to -0.166	**-0.344**	-0.479 to -0.210
Restricted Activity	-0.096	-0.248 to 0.056	-0.120	-0.260 to 0.020	-0.067	-0.223 to 0.088
**Risk Avoidance Domain**	-0.207	-0.333 to -0.081	**-0.452**	-0.583 to -0.321	**-0.356**	-0.486 to -0.226
Individual Risk Avoidance	-0.170	-0.293 to -0.046	-0.295	-0.449 to -0.141	-0.158	-0.307 to -0.010
Threats to Achievement	-0.197	-0.329 to -0.065	**-0.483**	-0.599 to -0.367	**-0.364**	-0.486 to -0.242
**Resilience Domain**	-0.109	-0.244 to 0.026	**-0.305**	-0.433 to -0.176	**-0.318**	-0.458 to -0.178
Family Involvement	0.094	-0.042 to 0.230	-0.115	-0.258 to 0.028	-0.203	-0.345 to -0.062
Physical Activity	-0.093	-0.226 to 0.040	-0.058	-0.196 to 0.081	-0.106	-0.262 to 0.051
Social Problem Solving	-0.205	-0.346 to -0.065	**-0.410**	-0.533 to -0.287	**-0.317**	-0.443 to -0.191
**Achievement Domain**	-0.205	-0.336 to -0.073	**-0.572**	-0.672 to -0.471	**-0.568**	-0.685 to -0.451
Academic Performance	-0.270	-0.415 to -0.125	**-0.639**	-0.736 to -0.543	**-0.541**	-0.664 to -0.418
Peer Relations	-0.073	-0.223 to 0.078	-0.284	-0.422 to -0.146	**-0.334**	-0.472 to -0.195

Abbreviations: CHIP-CE, Child Health and Illness Profile-Child Edition; ADHD-RS, Attention Deficit/Hyperactivity Disorder Rating Scale; CI, confidence interval; r, Pearson's correlation coefficient

Correlations larger than 0.3 were marked in bold to improve readability.

**Table 9:** Correlation between Child Health and Illness Profile-Child Edition and ADHD-Rating Scale hyperactive/impulsive subscore, by age groups based on data of all 5 trials.

CHIP-CE	Baseline		Endpoint		Change from baseline	
*Children n = 570-609*	r	95% CI	r	95% CI	r	95% CI
**Total Score**	-0.295	-0.365 to -0.224	**-0.482**	-0.545 to -0.419	**-0.478**	-0.541 to -0.414
**Satisfaction Domain**	-0.005	-0.092 to 0.082	-0.191	-0.269 to -0.113	-0.278	-0.357 to -0.198
Satisfaction with Health	0.077	-0.013 to 0.167	-0.092	-0.171 to -0.014	-0.189	-0.275 to -0.104
Satisfaction with Self	-0.093	-0.173 to -0.013	-0.261	-0.338 to -0.185	**-0.305**	-0.381 to -0.228
**Comfort Domain**	-0.154	-0.232 to -0.075	-0.253	-0.328 to -0.179	**-0.326**	-0.394 to -0.258
Physical Comfort	-0.012	-0.091 to 0.068	-0.039	-0.114 to 0.037	-0.129	-0.207 to -0.052
Emotional Comfort	-0.249	-0.319 to -0.179	**-0.373**	-0.444 to -0.302	**-0.402**	-0.466 to -0.337
Restricted Activity	0.018	-0.068 to 0.105	-0.035	-0.117 to 0.046	-0.074	-0.147 to -0.000
**Risk Avoidance Domain**	**-0.524**	-0.573 to -0.475	**-0.613**	-0.670 to -0.557	**-0.522**	-0.585 to -0.459
Individual Risk Avoidance	**-0.478**	-0.530 to -0.425	**-0.507**	-0.571 to -0.444	**-0.389**	-0.468 to -0.310
Threats to Achievement	**-0.478**	-0.531 to -0.425	**-0.587**	-0.643 to -0.531	**-0.502**	-0.566 to -0.437
**Resilience Domain**	-0.032	-0.107 to 0.043	-0.259	-0.335 to -0.183	-0.163	-0.247 to -0.079
Family Involvement	-0.026	-0.100 to 0.047	-0.179	-0.256 to -0.101	-0.128	-0.206 to -0.051
Physical Activity	0.174	0.096 to 0.252	-0.057	-0.138 to 0.024	-0.018	-0.095 to 0.058
Social Problem Solving	-0.163	-0.244 to -0.082	-0.263	-0.341 to -0.184	-0.168	-0.265 to -0.070
**Achievement Domain**	-0.196	-0.270 to -0.121	**-0.412**	-0.482 to -0.342	**-0.413**	-0.486 to -0.340
Academic Performance	-0.089	-0.162 to -0.015	**-0.361**	-0.436 to -0.285	**-0.376**	-0.453 to -0.299
Peer Relations	-0.210	-0.286 to -0.134	-0.295	-0.369 to -0.221	-0.287	-0.368 to -0.207
***Adolescents n = 165-181***						
**Total Score**	-0.347	-0.490 to -0.203	-0.481	-0.595 to -0.368	-0.436	-0.566 to -0.306
**Satisfaction Domain**	-0.041	-0.189 to 0.107	-0.143	-0.289 to 0.004	-0.219	-0.382 to -0.056
Satisfaction with Health	0.008	-0.141 to 0.157	-0.080	-0.228 to 0.067	-0.108	-0.267 to 0.050
Satisfaction with Self	-0.078	-0.216 to 0.059	-0.178	-0.322 to -0.034	-0.269	-0.422 to -0.115
**Comfort Domain**	-0.208	-0.349 to -0.067	-0.297	-0.429 to -0.166	-0.286	-0.428 to -0.144
Physical Comfort	-0.049	-0.177 to 0.080	-0.064	-0.213 to 0.085	-0.129	-0.273 to 0.016
Emotional Comfort	-0.302	-0.441 to -0.164	-0.426	-0.540 to -0.312	-0.371	-0.496 to -0.246
Restricted Activity	-0.005	-0.144 to 0.135	-0.079	-0.228 to 0.069	-0.049	-0.209 to 0.110
**Risk Avoidance Domain**	-0.568	-0.671 to -0.466	-0.593	-0.701 to -0.485	-0.359	-0.492 to -0.226
Individual Risk Avoidance	-0.474	-0.596 to -0.352	-0.484	-0.621 to -0.347	-0.181	-0.328 to -0.035
Threats to Achievement	-0.531	-0.633 to -0.430	-0.570	-0.673 to -0.468	-0.345	-0.476 to -0.215
**Resilience Domain**	-0.181	-0.322 to -0.040	-0.235	-0.370 to -0.100	-0.294	-0.432 to -0.156
Family Involvement	-0.085	-0.215 to 0.046	-0.198	-0.324 to -0.072	-0.181	-0.328 to -0.035
Physical Activity	0.091	-0.051 to 0.233	0.059	-0.083 to 0.201	-0.071	-0.210 to 0.068
Social Problem Solving	-0.292	-0.422 to -0.161	-0.311	-0.454 to -0.168	-0.309	-0.434 to -0.185
**Achievement Domain**	-0.233	-0.377 to -0.089	-0.472	-0.583 to -0.362	-0.471	-0.595 to -0.347
Academic Performance	-0.097	-0.244 to 0.050	-0.495	-0.603 to -0.388	-0.421	-0.557 to -0.284
Peer Relations	-0.244	-0.383 to -0.104	-0.264	-0.406 to -0.123	-0.282	-0.410 to -0.154

Abbreviations: CHIP-CE, Child Health and Illness Profile-Child Edition; ADHD-RS, Attention Deficit/Hyperactivity Disorder Rating Scale; CI, confidence interval; r, Pearson's correlation coefficient

Correlations larger than 0.3 were marked in bold to improve readability.

### 3.5 Differences in correlations between ADHD-RS and CHIP-CE scores, across age groups

No substantial age differences with respect to the correlations between ADHD-RS and CHIP-CE scores were found. However, in some cases, a trend for age differences in the correlations was observed. Figure 2 and 3 show the correlation between ADHD-RS total score and CHIP-CE, by age group.

## 4. Discussion

This meta-analysis must be seen in the broader context of previous research on Health-Related Quality of Life (HR-QoL) in children and adolescents with ADHD [45]. Several studies have investigated HR-QoL in these patients. These studies have shown robust negative effects on HR-QoL as reported both by parents and in patient self-reports. However, children with ADHD tend to rate their own HR-QoL less negatively than their parents and do not always see themselves as functioning less well than healthy controls [6]. More severe symptoms and greater impairment predict poorer HR-QoL. Evidence is increasing that HR-QoL improves with effective treatment, both with psychostimulants and with atomoxetine, but most treatment studies have had relatively short follow-up periods [6].

In comparing children and adolescents with ADHD, this meta-analysis investigated three different aspects: the evaluation of HR-QoL at baseline, the association between HR-QoL and ADHD core symptoms, and the treatment effect of atomoxetine on HR-QoL. The first two aspects were based on all 5 studies, whilst the treatment effect could only be evaluated in the 3 placebo-controlled trials.

In the population of the five studies, gender distribution was similar across age groups. As the studies were not designed to include the same proportion of boys across different age-groups, this finding is surprising. Usually one would assume that there would be a larger proportion of boys in a sample of children compared to a sample of adolescents. This could be due to the composition of samples in clinical trials as opposed to epidemiological samples.

Analyzing the ADHD-RS in the present post-hoc analysis, children had significantly higher hyperactive/impulsive sub-scores and total scores compared with adolescents at baseline. This finding is in line with previous literature regarding the differences in symptom patterns across age groups. Specifically, hyperactive/impulsive symptoms show a definite decline over time, while inattentive symptoms may become even more pronounced during adolescence [3,29-32]. In our sample, adolescents showed numerically higher inattentive sub-scores, although the difference in scores did not reach statistical significance and are unlikely to be clinically relevant. However, as the hyperactivity/impulsivity issues decrease, the relative importance of the inattention problems may increase. These findings need to put into perspective. Goodman et al 2010 [46] showed that ADHD-RS total score of 38.7 corresponded to moderately ill patients and 45.5 corresponded to markedly ill patients as measured by the CGI-S. Unfortunately, such data is lacking for the sub-scores of the ADHD-RS.

Baseline impairments in HR-QoL as measured by the CHIP-CE were seen on several dimensions (e.g., Satisfaction with Self, Threats to Achievement, and Academic Performance) in both age groups. Previous studies consistently reported on remarkable impairments in HR-QoL among children and adolescents with ADHD, especially in the emotional, behavioral, and achievement aspects [6]. Similarly, in our meta-analysis clinically relevant impairments were found in the Risk Avoidance and Achievement domains (and in their sub-domains), in the Emotional Comfort and in the Satisfaction with Self sub-domains as well as Family involvement and Social Problem Solving. Adolescents were generally more impaired, compared with children, in the Satisfaction with Self sub-domain, the Family Involvement sub-domain and in the Achievement domain, while children were more impaired on the Emotional Comfort sub-domain. It may be that inter-family relationships, cooperation with family members, self-satisfaction, and academic performance are more sensitive areas of life in an adolescent compared to a child (especially in the lower age-range, 6-7 years), and that ADHD symptoms might have a more pronounced effect on these domains among adolescents relative to children.

The baseline correlations between the CHIP-CE and ADHD-RS scores indicated a consistent, small-to-moderate negative correlation between the core symptoms of ADHD and HR-QoL in both age groups without substantial age differences. This finding provides additional insight into the broad effect of ADHD symptoms. However, it should be noted that these correlations do not fully explain the background of the impaired HR-QoL in children and adolescents with ADHD. Besides the core symptoms (as measured by the ADHD-RS), other factors might play a role in the observed HR-QoL impairments. For example, comorbidities such as oppositional defiant disorder (ODD), conduct disorder (CD), anxiety, and depression were found to increase impairment and decrease HR-QoL in children and adolescents with ADHD as measured by the CHIP-CE in a cross-sectional analysis of observational data [47]. This may explain the low to moderate correlation between ADHD core symptoms and HR-QoL in this meta-analysis. However, in order to analyze differential effects between children and adolescents in terms of factors influencing the impairment of HR-QoL, an even larger sample size would be required.

Based on our analysis, atomoxetine was effective in improving certain HR-QoL dimensions in both age groups. This finding is in line with several previous studies [15,18-25,48]. Our results indicate that adolescents might benefit more from atomoxetine treatment than children with regard to improvement in the Risk Avoidance domain and Threats to Achievement sub-domain. It must be taken into account that the sample size of adolescents in these studies was rather low, which may have prevented some of the observed therapeutic effects from reaching statistical significance (e.g. in the Achievement domain).

In both age groups, correlations between the ADHD core symptoms and the HR-QoL were small to moderate at endpoint and with regard to the change from baseline. There was no substantial age effect on the correlations, except for a clear trend in the Risk Avoidance domain and sub-domains. Specifically, the correlations between the ADHD-RS scores (both sub-scores and total score) and the Risk Avoidance domain and sub-domains were smaller in adolescents with regard to change from baseline. This reduction in the strength of the correlation between changes in core symptoms and HR-QoL may indicate a slight detachment from the primary therapeutic effect of atomoxetine on core symptoms, especially when taking into account that atomoxetine showed the highest effect sizes in improving HR-QoL in the Risk Avoidance domain and sub-domains. Our findings regarding the low and moderate correlations between core symptoms and HR-QoL (and the small correlations found in several instances regarding change from baseline after treatment), warrant further investigation to determine more precisely which additional factors contribute to the overall impairment in ADHD beyond core symptoms, and which particular factors have an adverse impact on the HR-QoL of the individuals and their family.

### 4.1 Limitations

The results of this meta-analysis need to be interpreted in light of a number of limitations. First, the samples of the five clinical trials showed heterogeneity in terms of cultural diversity, history of stimulant medication, and comorbidity. For example, the patients were from five different countries, where both public opinion on ADHD and approaches to treatment by physicians vary considerably. Such differences in terms of cultural diversity could have had an impact on the evaluation both of core symptoms as measured with the ADHD-RS, and health-related quality of life as measured with the CHIP-CE. Moreover, the pooled sample size of the adolescent treatment group from the three placebo-controlled trials was rather small, and thus, effect size estimations have to be interpreted with caution.

Second, drug history of the patients was mostly unknown (with the exception of Studies 2 and 4, where one of the inclusion criteria was that the patients had to be treatment-naïve): this could have introduced some variability in the evaluation of treatment efficacy. It has been already suggested in the literature that medication-naïve patients show better improvement [19]. However, in Study 4, authors reported a lack of interaction between the treatment group (atomoxetine or standard care) and whether patients had been previously treated with medication for their ADHD, indicating that the treatment effect was similar for both groups of patients (treatment-naïve or not) in terms of improving CHIP-CE total score [23]. Unfortunately, power to detect such interactions is generally low and further research is needed to obtain more information on treatment effect modifiers to ultimately tailor the medication to the individual patient.

Third, 8-12 weeks of follow-up might have been too brief for the evaluation of the improvement of HR-QoL. Though the findings of Perwien et al. [19] indicate that the treatment effect of atomoxetine with regard to the improvement in HR-QoL can be detected after 7 to 8 weeks of treatment with atomoxetine, long-term studies are warranted in this regard: primary symptoms might change significantly within 3 months, but the consequences, at least in part, might need a longer period for improvement and/or stabilization. This needs to be taken into account when evaluating the clinical impact of the differences. The developers of the CHIP-CE have proposed that a threshold of 0.6 standard deviations is clinically meaningful [49].

Fourth, in all studies, parents were the source of information on both core symptoms of ADHD and HR-QoL. This might have influenced the results in the sense that the parents and patients might have provided different responses, especially when evaluating adolescents. The views of the young people themselves, however, need to be sought in addition to parent reports, as the patient perspective reflects the subjective well-being of these children and adolescents and takes into account their autonomy as individuals [45].

An additional limitation that introduces a difficulty in interpreting our results is that HR-QoL is a construct that, to date has not yet been well-defined. Hence, measuring this construct is still a challenge, as are all measurements of subjectively perceived psychological constructs [6]. Although the CHIP-CE was validated and standardized on a large community sample of children and adolescents, it cannot be assured that CHIP-CE really reflects and captures all the relevant aspects of HR-QoL with regard to the evaluation of the broad impact of ADHD on the individual's life.

### 4.2 Strengths

This meta-analysis also had several strengths. Most importantly, the sample size was large. Secondly, three of the five studies were placebo-controlled. Thirdly, the analysis was based on individual patient data rather than publication-based meta-analysis. Fourthly, the inclusion and exclusion criteria of the five studies included in the meta-analysis [47] were very similar, resulting in a fairly homogeneous sample in terms of patient characteristics. Finally, the meta-analysis included patient reported HR-QoL outcomes as a secondary endpoint. Thus, the analysis can be considered an important contribution to the body of data on the relationship between outcomes in terms of ADHD core symptoms and HR-QoL outcomes based on closely monitored clinical trials rather than cross-sectional (or observational) studies.

## 5 Conclusion

Overall, this meta-analysis found that, compared with children, adolescents with ADHD were somewhat more impaired at baseline, in regard to some domains of HR-QoL as measured by the CHIP-CE. Impairments were seen in the Risk Avoidance and Achievement domains and their sub-domains as well as in the sub-domains Emotional Comfort, Satisfaction with Self, Family Involvement, and Social Problem Solving, both in children and adolescents. Atomoxetine was generally shown to be effective in improving certain aspects of HR-QoL as reflected by the CHIP-CE. In the Risk Avoidance domain and Threats to Achievement sub-domain, there was a significant age effect with better efficacy seen in adolescents. Correlations between ADHD core symptoms and HR-QoL at baseline and for change from baseline to endpoint were small to moderate, suggesting that next to the effect of core symptoms, other factors might play a role in the background of the observed impairments in HR-QoL. Further studies are needed to investigate the long-term effects of atomoxetine on HR-QoL, as well as to develop more specific tools in the assessment of the effect of ADHD treatments on HR-QoL in children and adolescents.

## Competing interests

The research was funded by Eli Lilly and Company. PMW, AS, RE, and NS are full-time employees and stakeholders of Eli Lilly. VH has received research grants and speaker honoraria from Eli Lilly and has served on several advisory boards for Eli Lilly.

## Authors' contributions

PMW, AS, and RE developed the meta-analysis on which this manuscript is based. All Authors participated in interpreting the data. PMW and AS drafted the manuscript. RE, NS and VH revised the manuscript for important intellectual content. All authors have read and approved the final version of the manuscript.
